# Supplementary figures and images for: A Noninvasive Imaging Toolbox Indicates Limited Therapeutic Potential of Conditionally Activated Macrophages in a Mouse Model of Multiple Organ Dysfunction
Source: Stem Cells Int. 2019 Apr 1;2019:7386954. doi: 10.1155/2019/7386954 (PMC6466849; doi:10.1155/2019/7386954)

## Slide 1
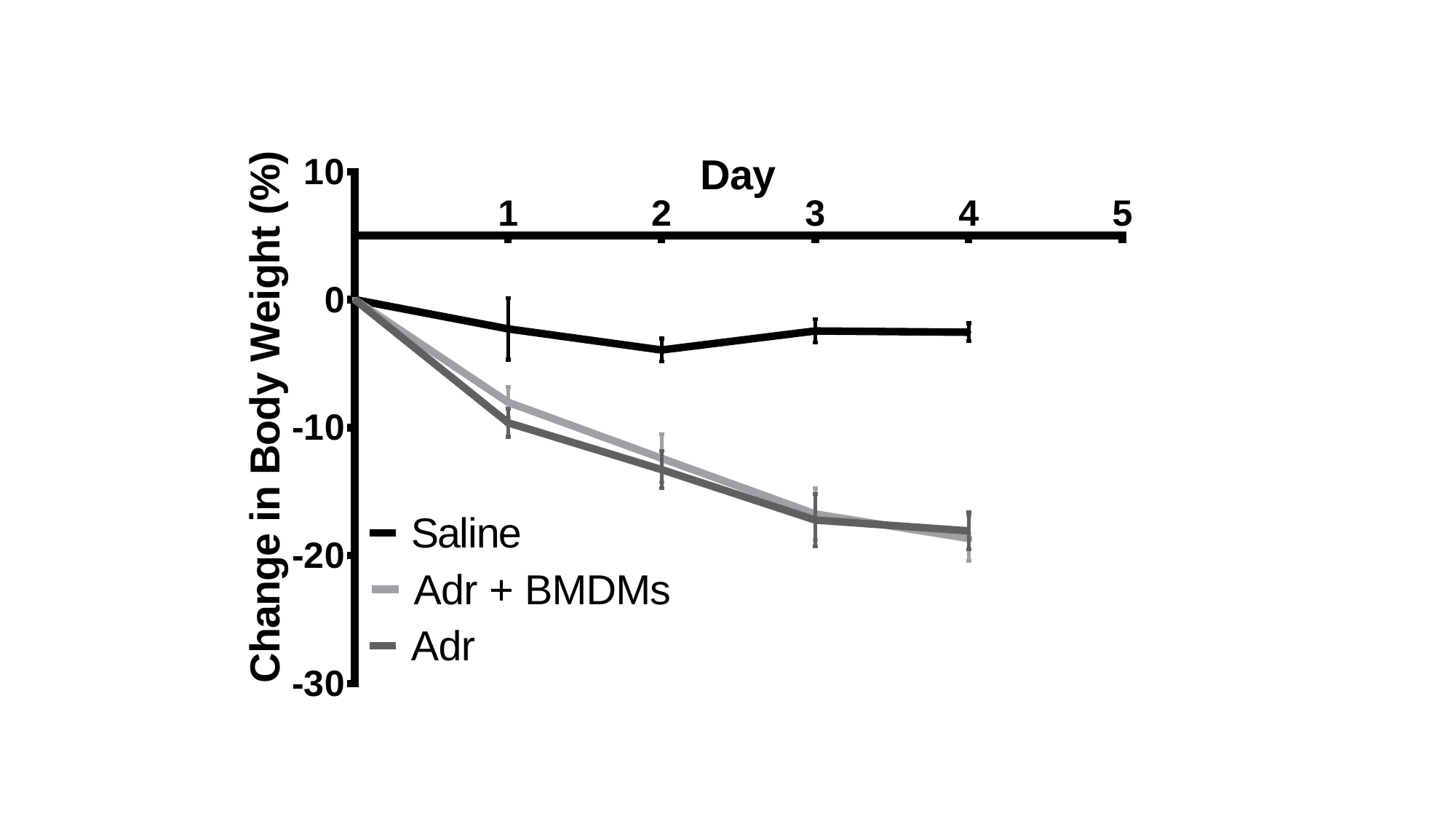

Supplement: Supplementary 1 — Supplementary Figure 1: changes in body weight of the mice over the study. [file 7386954.f1.pptx]

## Slide 1
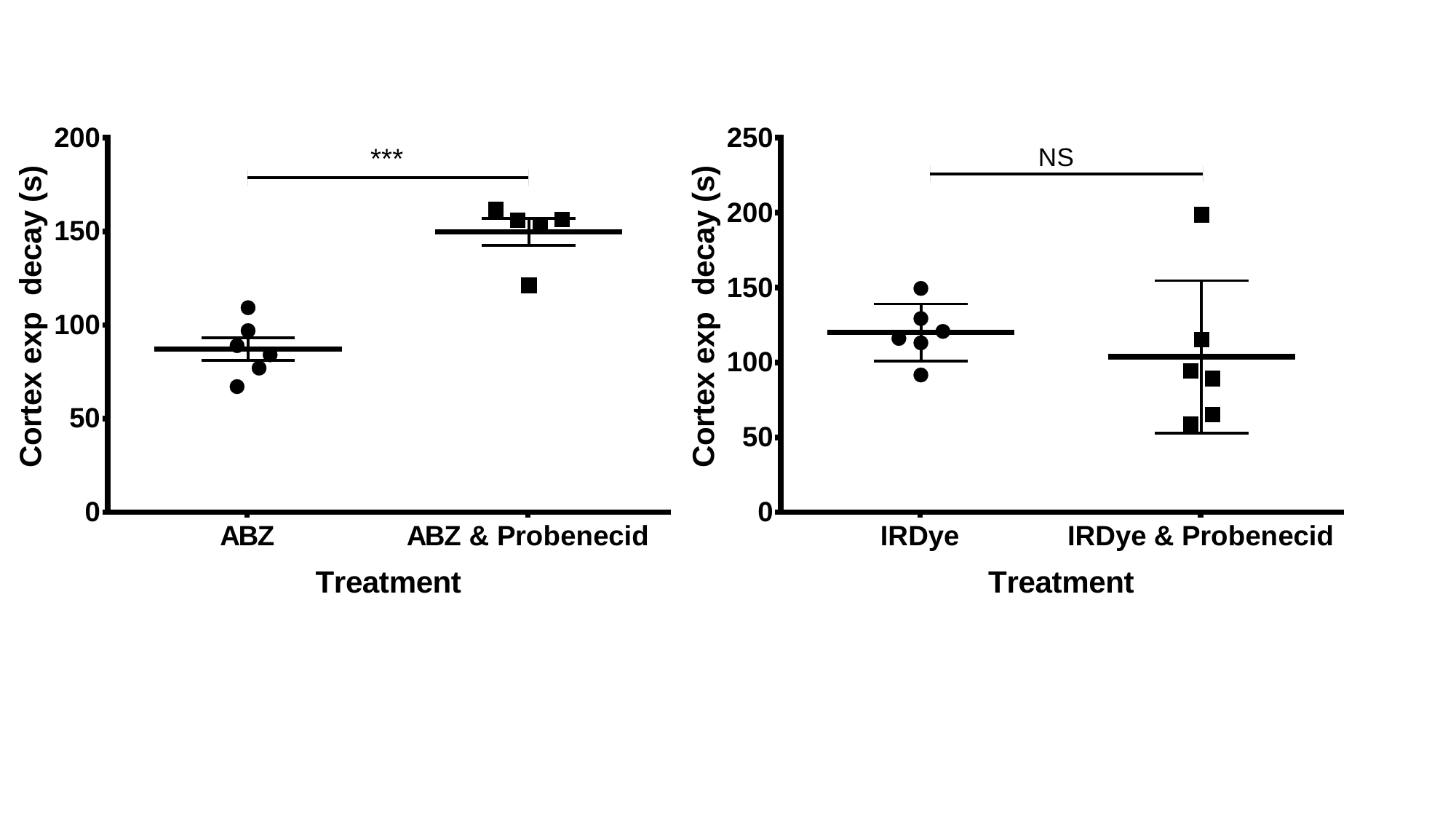

Supplement: Supplementary 2 — Supplementary Figure 2: the effect of probenecid on IRDye clearance in mice. [file 7386954.f2.pptx]

## Slide 1
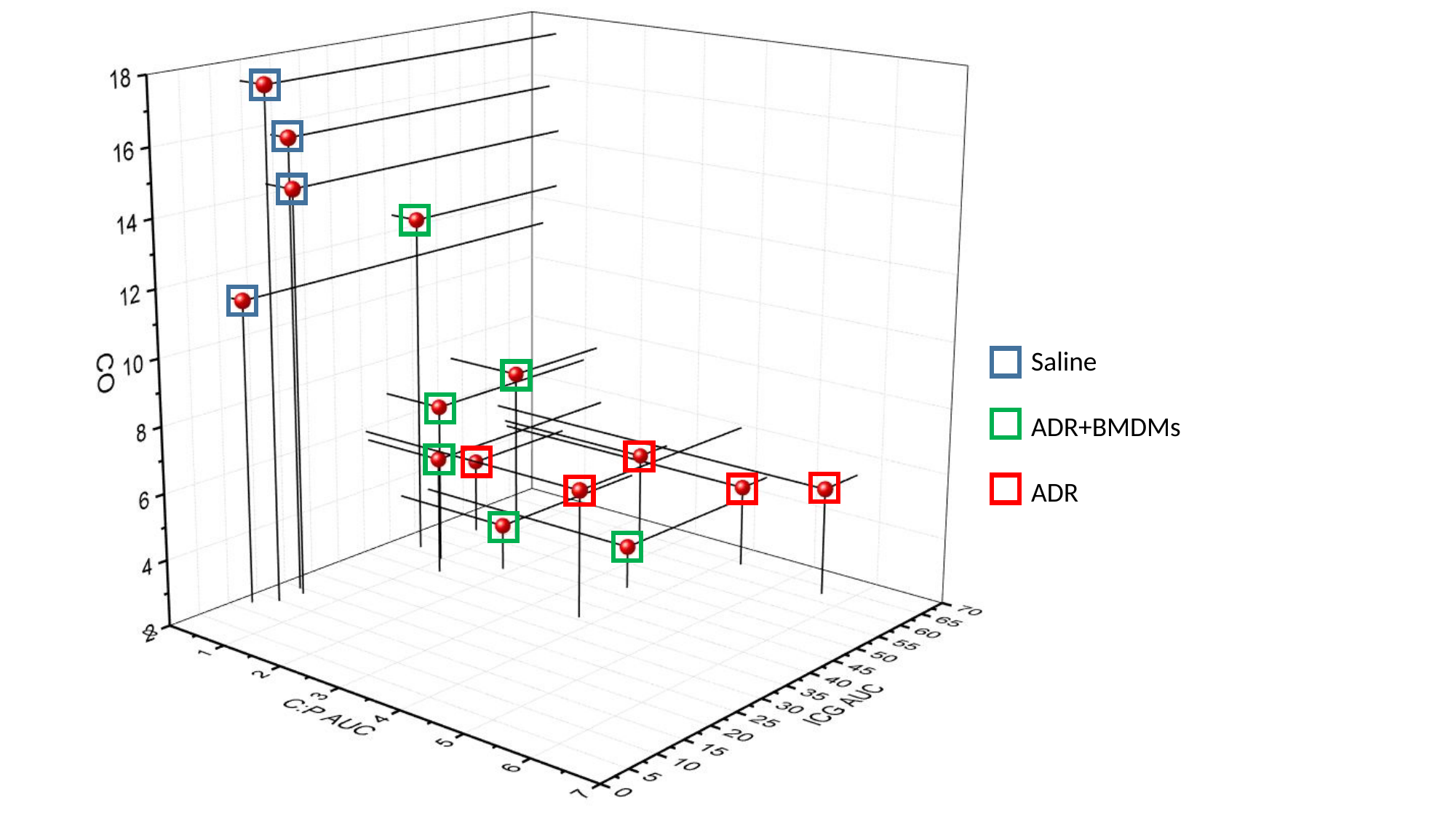

Saline
ADR+BMDMs
ADR

Supplement: Supplementary 3 — Supplementary Figure 3: the correlation between cardiac output, C:P AUC, and ICG AUC in mice in the study. [file 7386954.f3.pptx]
