# Supplementary material for: A Noninvasive Imaging Toolbox Indicates Limited Therapeutic Potential of Conditionally Activated Macrophages in a Mouse Model of Multiple Organ Dysfunction
Source: Stem Cells Int. 2019 Apr 1;2019:7386954. doi: 10.1155/2019/7386954 (PMC6466849; doi:10.1155/2019/7386954)
Supplement: Supplementary 4 — Supplementary Table 1: imaging and biomarker data. [file 7386954.f4.pptx]

## Slide 1
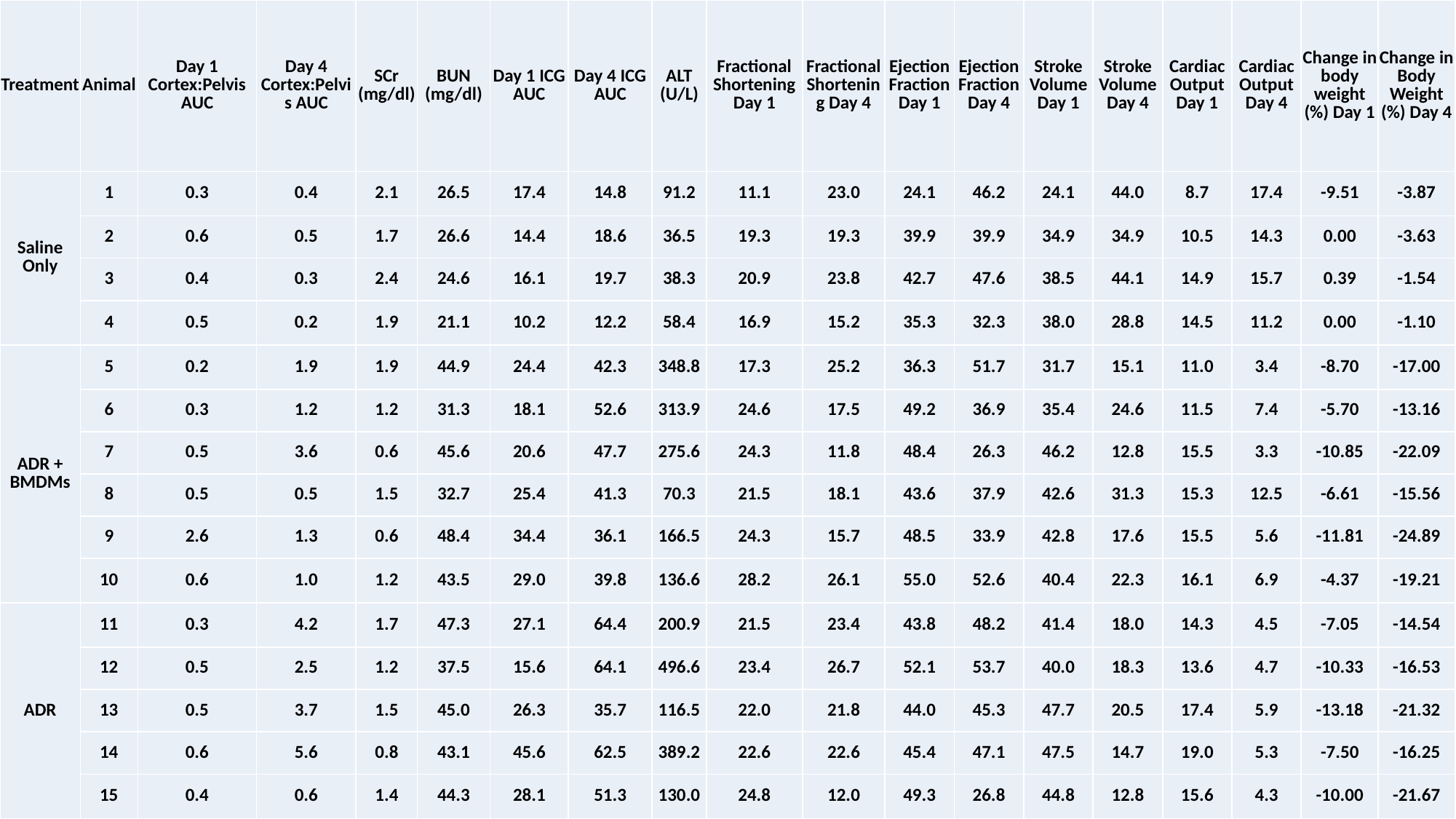

| Treatment | Animal | Day 1 Cortex:Pelvis AUC | Day 4 Cortex:Pelvis AUC | SCr (mg/dl) | BUN (mg/dl) | Day 1 ICG AUC | Day 4 ICG AUC | ALT (U/L) | Fractional Shortening Day 1 | Fractional Shortening Day 4 | Ejection Fraction Day 1 | Ejection Fraction Day 4 | Stroke Volume Day 1 | Stroke Volume Day 4 | Cardiac Output Day 1 | Cardiac Output Day 4 | Change in body weight (%) Day 1 | Change in Body Weight (%) Day 4 |
| --- | --- | --- | --- | --- | --- | --- | --- | --- | --- | --- | --- | --- | --- | --- | --- | --- | --- | --- |
| Saline Only | 1 | 0.3 | 0.4 | 2.1 | 26.5 | 17.4 | 14.8 | 91.2 | 11.1 | 23.0 | 24.1 | 46.2 | 24.1 | 44.0 | 8.7 | 17.4 | -9.51 | -3.87 |
| | 2 | 0.6 | 0.5 | 1.7 | 26.6 | 14.4 | 18.6 | 36.5 | 19.3 | 19.3 | 39.9 | 39.9 | 34.9 | 34.9 | 10.5 | 14.3 | 0.00 | -3.63 |
| | 3 | 0.4 | 0.3 | 2.4 | 24.6 | 16.1 | 19.7 | 38.3 | 20.9 | 23.8 | 42.7 | 47.6 | 38.5 | 44.1 | 14.9 | 15.7 | 0.39 | -1.54 |
| | 4 | 0.5 | 0.2 | 1.9 | 21.1 | 10.2 | 12.2 | 58.4 | 16.9 | 15.2 | 35.3 | 32.3 | 38.0 | 28.8 | 14.5 | 11.2 | 0.00 | -1.10 |
| ADR + BMDMs | 5 | 0.2 | 1.9 | 1.9 | 44.9 | 24.4 | 42.3 | 348.8 | 17.3 | 25.2 | 36.3 | 51.7 | 31.7 | 15.1 | 11.0 | 3.4 | -8.70 | -17.00 |
| | 6 | 0.3 | 1.2 | 1.2 | 31.3 | 18.1 | 52.6 | 313.9 | 24.6 | 17.5 | 49.2 | 36.9 | 35.4 | 24.6 | 11.5 | 7.4 | -5.70 | -13.16 |
| | 7 | 0.5 | 3.6 | 0.6 | 45.6 | 20.6 | 47.7 | 275.6 | 24.3 | 11.8 | 48.4 | 26.3 | 46.2 | 12.8 | 15.5 | 3.3 | -10.85 | -22.09 |
| | 8 | 0.5 | 0.5 | 1.5 | 32.7 | 25.4 | 41.3 | 70.3 | 21.5 | 18.1 | 43.6 | 37.9 | 42.6 | 31.3 | 15.3 | 12.5 | -6.61 | -15.56 |
| | 9 | 2.6 | 1.3 | 0.6 | 48.4 | 34.4 | 36.1 | 166.5 | 24.3 | 15.7 | 48.5 | 33.9 | 42.8 | 17.6 | 15.5 | 5.6 | -11.81 | -24.89 |
| | 10 | 0.6 | 1.0 | 1.2 | 43.5 | 29.0 | 39.8 | 136.6 | 28.2 | 26.1 | 55.0 | 52.6 | 40.4 | 22.3 | 16.1 | 6.9 | -4.37 | -19.21 |
| ADR | 11 | 0.3 | 4.2 | 1.7 | 47.3 | 27.1 | 64.4 | 200.9 | 21.5 | 23.4 | 43.8 | 48.2 | 41.4 | 18.0 | 14.3 | 4.5 | -7.05 | -14.54 |
| | 12 | 0.5 | 2.5 | 1.2 | 37.5 | 15.6 | 64.1 | 496.6 | 23.4 | 26.7 | 52.1 | 53.7 | 40.0 | 18.3 | 13.6 | 4.7 | -10.33 | -16.53 |
| | 13 | 0.5 | 3.7 | 1.5 | 45.0 | 26.3 | 35.7 | 116.5 | 22.0 | 21.8 | 44.0 | 45.3 | 47.7 | 20.5 | 17.4 | 5.9 | -13.18 | -21.32 |
| | 14 | 0.6 | 5.6 | 0.8 | 43.1 | 45.6 | 62.5 | 389.2 | 22.6 | 22.6 | 45.4 | 47.1 | 47.5 | 14.7 | 19.0 | 5.3 | -7.50 | -16.25 |
| | 15 | 0.4 | 0.6 | 1.4 | 44.3 | 28.1 | 51.3 | 130.0 | 24.8 | 12.0 | 49.3 | 26.8 | 44.8 | 12.8 | 15.6 | 4.3 | -10.00 | -21.67 |
